# Understanding health-related behavior among adolescents living with HIV in Lima, Peru

**DOI:** 10.1186/s12887-019-1773-3

**Published:** 2019-10-31

**Authors:** Carly A. Rodriguez, Emiliano Valle, Jerome Galea, Milagros Wong, Lenka Kolevic, Maribel Muñoz, Leonid Lecca, Molly F. Franke

**Affiliations:** 1 Department of Global Health and Social Medicine, Harvard Medical School, 641 Huntington Avenue, Boston, MA 02115 USA; 2 Socios En Salud Sucursal Peru, Ave Merino Reyna 575, Carabayllo, Lima 6, Peru; 3 School of Social Work, University of South Florida, 13301 Bruce B Downs Blvd, MHC 1416 A, Tampa, Florida, 33612-3807 USA; 4 Infectious Disease, Instituto Nacional de Salud del Niño, Ave Brasil 600, Breña, 15083 Lima, Peru

**Keywords:** Health behavior, Peru, Adolescent health, Adolescent behavior, Sexual activity, Adherence

## Abstract

**Background:**

The global HIV burden among adolescents ages 10–19 is growing. This population concurrently confronts the multifaceted challenges of adolescence and living with HIV. With the goal of informing future interventions tailored to this group, we assessed sexual activity, HIV diagnosis disclosure, combination antiretroviral therapy (cART) adherence, and drug use among adolescents living with HIV (ALHIV) in Lima, Peru.

**Methods:**

Adolescents at risk or with a history of suboptimal cART adherence completed a self-administered, health behaviors survey and participated in support group sessions, which were audio recorded and used as a qualitative data source. Additionally, we conducted in-depth interviews with caregivers and care providers of ALHIV. Thematic content analysis was performed on the group transcripts and in-depth interviews and integrated with data from the survey to describe adolescents’ health related behaviors.

**Results:**

We enrolled 34 ALHIV, of which 32 (14 male, 18 female, median age 14.5 years) completed the health behavior survey. Nine (28%) adolescents reported prior sexual intercourse, a minority of whom (44%) reported using a condom. cART adherence was highest in the 10–12 age group with 89% reporting ≤2 missed doses in the last month, compared to 36% in adolescents 13 years or older. Over 80% of adolescents had never disclosed their HIV status to a friend or romantic partner. Adolescents, caregivers, and health service providers described sexual health misinformation and difficulty having conversations about sexual health and HIV.

**Conclusions:**

In this group of ALHIV, adherence to cART declined with age and condom use among sexually active adolescents was low. Multifactorial interventions addressing sexual health, gaps in HIV-related knowledge, and management of disclosure and romantic relationships are urgently needed for this population.

## Background

Scientific advances and expanded access to combination antiretroviral therapy (cART) over the last decade have led to major improvements in the survival of children perinatally-infected with HIV. This, coupled with sustained behavioral transmission, has led to a 28% increase in the number of adolescents living with HIV (ALHIV) since 2005, [1] and an estimated 1.8 million ALHIV ages 10–19 globally [2]. Understanding the health-related behaviors and beliefs of adolescents living with HIV (ALHIV) is critical for the design of health interventions for this vulnerable population.

Adolescence is a developmental phase characterized by profound physical growth and cognitive, social, emotional, and sexual development. Adolescents begin romantic relationships and often have condomless sex [3]. Experimentation with alcohol and drugs is common as adolescents search for peer acceptance [4]. These behaviors, among others, are characteristic of development during youth and can place adolescents at increased risk for acquiring and transmitting infections like HIV [3]. ALHIV must additionally confront the challenges of adhering to cART and a schedule of routine medical appointments, disclosing the diagnosis to friends and romantic partners, and HIV-related stigma [5]. These hurdles often manifest in adverse social and medical consequences including a greater difficulty managing relationships, [5–7] lower cART adherence, [8] and higher mortality [9, 10].

Despite the unique challenges faced by ALHIV, they are often overlooked and underrepresented in research, compromising the applicability of study findings. Much of the available research in HIV among adolescents is from North America and Sub-Saharan Africa, which limits its generalizability to other settings [11, 12]. Consequently, effective evidence-based interventions for this group are lacking, [5] especially in Latin America where half of new infections occur among adolescents and young adults [13]. Two systematic reviews of cART adherence interventions for ALHIV identified no interventions in Latin America [8, 14]. Only 2% of ALHIV in a recent global cohort analysis were from countries of Latin America and the Caribbean [15]. Of the research among ALHIV in Latin America, the overwhelming majority of evidence originates from studies conducted in Brazil [16–18]. Culturally-tailored interventions for ALHIV must be developed and rigorously evaluated to meet this population’s diverse needs.

The objective of this study was to gain an understanding of health-related behaviors among ALHIV in Lima, Peru. Adolescents provided quantitative data on behaviors such as sexual activity, cART adherence, disclosure and substance use, which was supplemented with qualitative data originating from the adolescents as well as health personnel and caregivers of ALHIV.

## Methods

### Study setting

We conducted our study in Peru, which has an estimated 70,000 people living with HIV [19]; of these, 20% are youth ages 15 to 24 years. The highest proportion of new HIV diagnoses in 2018 occurred in youth and young adults ages 18 to 29 years: 43% of new diagnoses in women and 50% of new diagnoses in men were among people ages 18 to 29 years [20]. While the majority of ALHIV in Peru are perinatally infected, [21] the number of new HIV diagnoses in older adolescents (15 to 19 years) increased by nearly 75% between 2009 to 2013 and 2014 to 2018 [20].

### Participants

We recruited 34 ALHIV ages 10–17 years residing in the Lima Metropolitan area to participate in a six-month psychosocial support group intervention. Eligible adolescents were: prescribed cART; receiving HIV care at the National Institute for Child Health (Instituto Nacional de Salud del Niño, (INSN)) in Lima; were aware of their HIV diagnosis; and were at high risk of cART non-adherence, as determined by their physician (due to a challenging social environment such as an unstable living situation or a history of suboptimal adherence). INSN is a national public sectoral referral hospital for children and hosts the largest pediatric HIV clinic in the country. INSN provides care for the largest number of children and adolescents living with HIV in Peru. ALHIV were identified as eligible for inclusion by their physician, and were approached to participate during a routine clinical encounter. The provider and a study staff member described the psychosocial support group intervention and study requirements. Adolescents were informed that their choice to participate in the study would not affect their standard of care (i.e., access to psychologist and/or social worker evaluation, referrals to specialty services as needed). Social support groups were not routinely available to adolescents as part of standard care before or after the intervention period.

We enrolled five health personnel and staff and ten caregivers of ALHIV to participate in in-depth interviews. Health personnel provided HIV-related care or services under the auspices of Peruvian Ministry of Health’s HIV Program at INSN and included a physician, nurse, psychologist, health educator, and peer counselor. Caregivers were purposefully selected to represent a variety of caregiver types, including five mothers, two non-family caregivers, two caregivers at a group home, and one non-mother family caregiver (a brother). Caregivers were approached by INSN clinicians to participate during their child’s routine clinic visits, but were not necessarily caregivers of the ALHIV who participated in the psychosocial support groups. Four (40%) caregivers also had their adolescent enrolled in the study.

### Data collection

We concurrently collected data from three sources: surveys completed by ALHIV, transcripts of psychosocial support groups for ALHIV, and in-depth interviews with health personnel and caregivers of ALHIV. The aim of the psychosocial support groups was to provide a venue for ALHIV to ask questions and engage in discussion about their questions and experiences, many of which focused on living with HIV. The psychosocial support groups were foremost an intervention; however, transcriptions from the sessions afforded a unique opportunity to learn about the experiences of ALHIV in this setting and were analyzed as a qualitative data source. Data collection occurred between June 2015 and December 2015.

#### Self-administered survey

Prior to initiation of the psychosocial support group intervention, adolescents completed an audio computer-assisted self-interview (ACASI) to assess HIV- and health-related behaviors. ACASI allows participants to listen to recorded questions and respond using a touch screen tablet, thereby reducing social-desirability response bias [22–24]. The survey included questions on cART adherence, diagnosis disclosure, and a subset of questions from the Youth Risk Behavior System Surveillance Scale, including sexual activity and substance use [25].

#### Psychosocial support group discussions

Adolescents participated in twelve bi-weekly psychosocial support group sessions over a six-month period. Three groups of adolescents were formed according to age: 10–12 years, 13–15 years, and 16–17 years. Psychosocial support group sessions, conducted in Spanish by a trained facilitator, lasted approximately 90 min and were audio recorded. The facilitators were women living with HIV who had received training in group facilitation by the study investigators and were unacquainted with the adolescents prior to initiation of the groups. After each session, a psychologist on the study team reviewed the audio-recording to ensure quality and provide feedback to the facilitators as needed. Because the primary objective of the support group was to foster an environment of mutual support for the adolescents, facilitators used a modified operative group approach [26]. Under this model, the participants themselves decided on the discussion topics with the facilitator helping to guide the exploration of the emergent theme among the participants, assure group norms (e.g., punctuality, confidentiality, respect), encourage participation by the group members, and correct factual inaccuracies. Because there were 12 sessions per age group, each with the same participants, some discussion topics reprised and the facilitator could also draw on earlier discussions of the same topics to encourage discussion. Adolescents and their accompanying caregiver were provided with lunch and reimbursement for transportation.

#### In-depth interviews

In-depth interviews lasting approximately 1 hour were conducted with health personnel and caregivers responsible for the care of ALHIV. Interviews were conducted in Spanish by study personnel trained in qualitative research methods. Study personnel followed a semi-structured guide and interviews were audio recorded. The interview guide included questions on HIV disclosure; stigma and discrimination; cART adherence; challenges to care; emotional and psychological issues; and romantic and sexual relationships.

### Data analysis

We calculated descriptive statistics from ACASI surveys and compared and contrasted qualitative data to gain a deeper understanding of the behaviors reported quantitatively. Audio recordings from psychosocial support groups and in-depth interviews were transcribed verbatim and loaded into Dedoose (version 7.0.23, SocioCultural Research Consultants, Los Angeles, CA), a web-based qualitative analysis software [27]. To become familiar with the content and to build codebooks of common themes, we (MW, a native Spanish speaker, and JG, a bilingual Spanish-English speaker) read the Spanish transcripts and coded them according to themes. We added themes that appeared during a second transcript reading and coding analysis and reconciled coding discrepancies and refined code definitions throughout. We extracted and reviewed excerpts of transcripts that were coded as relating to issues explored in the quantitative interview: substance use, sexual activity, diagnosis disclosure, and adherence. Selected quotes were translated into English and back-translated into Spanish to ensure translation fidelity. We noted common themes that emerged and highlighted representative or informative quotes.

### Human subjects

Study materials were reviewed and approved by research ethics committees at the Peruvian National Institutes of Child Health (Instituto Nacional de Salud del Niño), Lima, Peru and Harvard Medical School, Boston, USA. We obtained written informed consent from adult health personnel. For adolescents, we obtained written informed assent from adolescents and written informed consent from a parent or biological family member; in the case a caregiver was not a biological family member, we followed guidelines provided by the ethics committee of INSN to obtain consent from a legal guardian.

## Results

We enrolled 34 adolescents, of whom 32 had quantitative survey data available. Nine (28%) adolescents were 10–12 years, 12 (38%) were 13–15 years, and 11 (34%) were 16–17 years (Table 1). Approximately half (18/32, 56%) of participants were female with each age group having roughly equal numbers of each sex (data not shown). Emergent themes that arose from the social support group discussions and in-depth interviews included adherence to cART, sex and relationships (including condom use, falling in love), disclosure of HIV status, and substance use.

**Table 1:** Participant characteristics (*N* = 32 unless noted otherwise)

Characteristic	N (%)
Age (years), median (IQR)	14.5 (12–16)
Female	18 (56%)
Grade	
3rd grade primary or less	3 (9%)
4th to 5th grade primary	3 (9%)
6th grade primary	4 (13%)
1st to 3rd grade secondary	14 (44%)
4th to 5th grade secondary	7 (22%)
Not in school or other	1 (3%)
Reported being bullied in school	7 (22%)
Reported being bullied electronically	4 (13%)
Grades (*N* = 28)	
A (15–16)	7 (25%)
B (13–14)	8 (29%)
C (11–12)	10 (36%)
D (10)	3 (11%)
E (<10)	0
Participates in clubs or activities at school	17 (53%)
Perceived school as important or very important for future	31 (97%)
Disclosed HIV status to at least one friend	6 (19%)
Number of friends disclosed HIV status to (*N* = 6)	
1–2	2 (33%)
3–10	2 (33%)
10–20	1 (17%)
>20	1 (17%)
Smoking	
Ever tried smoking	9 (28%)
Days smoked cigarettes in the last 30 days (*N* = 9)	
0 days	5 (56%)
1 or 2 days	4 (44%)
≥3 days	0
During the last 30 days, number of cigarettes smoked on days when smoked (N = 9)	
None	0
Less than one cigarette per day	8 (89%)
1 cigarette per day	1 (11%)
≥2 cigarettes per day	0
Alcohol use	
Ever had alcohol (more than one or two sips)	11 (34%)
Age when first drank more than one or two sips of alcohol (*N* = 10)	
8 years or younger	0
9 or 10 years	1 (9%)
11 or 12 years	0
13 or 14 years	3 (27%)
15 or 16 years	5 (45%)
17 or 18 years	1 (9%)
During the last 30 days, number of days drank at least one alcoholic beverage (*N* = 11)	
0 days	6 (55%)
1 or 2 days	5 (45%)
≥3 days	0
During last 30 days, number of days drank 5 or more alcoholic beverages in a row in roughly 2 hours (*N* = 11)	
0 days	7 (64%)
1 or 2 days	4 (36%)
≥3 days	0
Drug use^a^	
Ever consumed drugs	1 (3%)
Number of days used marijuana in last 30 days (*N* = 1)	
0 times	0
1 or 2 times	1 (100%)
≥3 times	0
Sexual activity	
Had sex	9 (28%)
Age at sexual debut (*N* = 9)	
≤11 years	0
12 years	1 (11%)
13 years or older	8 (89%)
Number of sexual partners (*N* = 9)	
1 person	6 (67%)
2 people	0
3 people	2 (22%)
4 people	1 (11%)
Used a condom during last time had sex (*N* = 9)	4 (44%)

^a^Drug use was defined as ever consuming: marijuana; cocaine paste or cocaine; inhaled “terokal” (vapors from solvents such as glue), the contents of an aerosol can, or other gases or sprays intending to get high; or other drugs

### Adherence

**Fig. 1:**
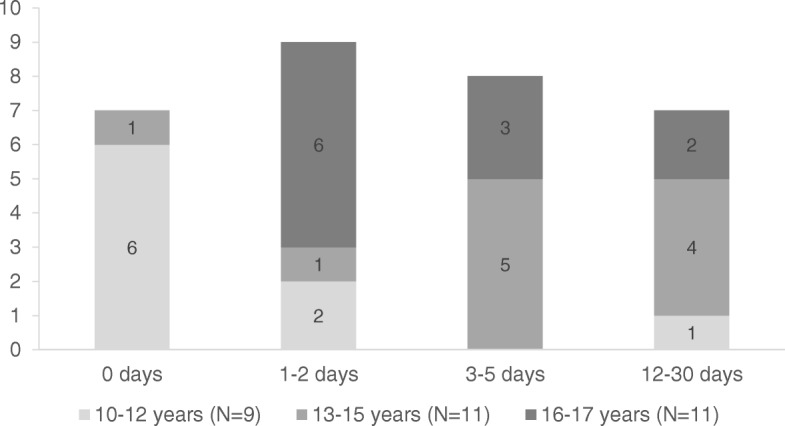
Reported number of days (of last 30) with at least one missed dose of cART (*N* = 31)

Figure 1 shows the number of days adolescents reported ≥1 missed dose of cART in the month prior to the survey by age group. The proportion of adolescents missing a dose for ≥3 days was greatest in the 13–15 year age group: 5 (45%) missed a dose on 3–5 days and 4 (36%) missed a dose on 12–30 days, compared to 3 (27%) and 2 (18%) of 16–17 year olds missing a dose on 3–5 and 12–30 days, respectively. Adherence among 10–12 year olds was higher with all but one reporting ≤2 missed doses in the last month. Barriers and facilitators to cART adherence in this study population have been reported previously [28].

### Sexual activity

**Fig. 2:**
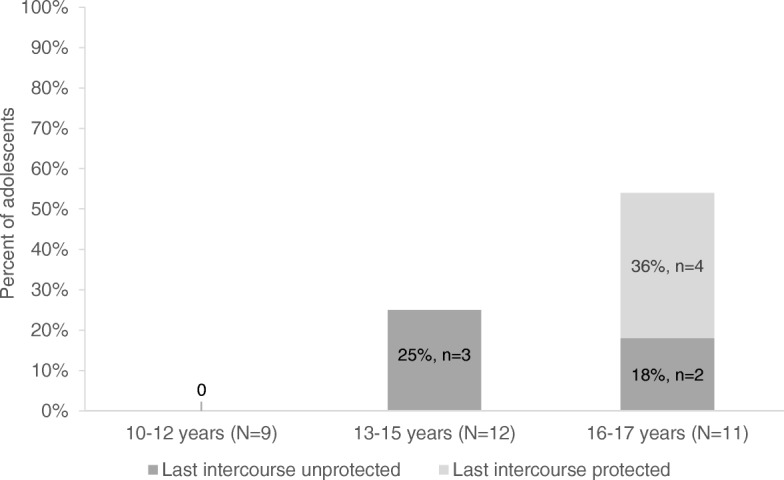
Percentage of adolescents reporting having ever had sex, by age group (*N* = 32)

Nine adolescents (28%), all at least 13 years, reported ever having sex. Of these, four (44%) reported using a condom during their last sexual intercourse (Table 1, Fig. 2). Misinformation related to sexual health was apparent in support group discussions around condom use:

Female (1): Seriously, they tell us that [condoms] supposedly protect against HIV, against AIDS, but they do it for publicity, so that people buy them, but in reality, it doesn’t protect, I was told. Because they say that the threads of the, of the … of that thing, what is it called? They aren’t small enough to block the virus from the, the infected person.

Female (1), 17 years.

Female (1): They taught me that the condom has to be … has to be rolled on.

Female (2): No! My aunt told me that you must inflate it.

Female (1), 17; Female (2), 17 years.

Qualitative data suggested inadequate communication between adolescents and caregivers regarding sexual health, and lack of access to accurate and continuous health information from other sources contributed to inadequate information on sexual health.

#### Inadequate communication between adolescents and caregivers

Caregivers reported multiple reasons for not discussing sexual health with the adolescents, including the perception that the topic was not relevant, a lack of confidence in how to effectively engage in these conversations, and a lack of resources to gain skills in discussing sexual health with their adolescents. The non-family caregiver of a 15-year old adolescent doubted the relevance of sexual health conversations and his ability to clearly convey information to his adolescent:

Caregiver (non-family): Well since I don’t see that much with girlfriends at home or hugging … so, only from afar really, I don’t take the time [to discuss it].

Interviewer: But he has had girlfriends [I mean]…you’ve seen that he has?

Caregiver (non-family): Umm yeah, I don’t think...I’ve just heard [about it]. It’s not that he’s had or that I have seen him hugging.

Interviewer: You haven’t spoken specifically with him to know what he did or....?

Caregiver (non-family): No.

Interviewer: And you’ve also not talked to him about condom use?

Caregiver (non-family): I think I did just once, and my friends also tell him things more clearly than I do.

Some caregivers expressed uncertainty in how to effectively engage their adolescents in conversations around sexual health and reported these conversations were met with resistance:

Caregiver (mother): [My daughter] doesn’t want to talk about that and it is precisely the reason why I wanted to talk to [the peer counselor]. I want to let her know about the sexual relationships they were talking about. And it just so happened that [at the health facility] they were giving an [educational] talk about this. And [the adolescents] should tell when they have sex. They should tell when they have a boyfriend, whether they use a condom or no condom … how does this [conversation] happen?

Interviewer: You’re the one who has to speak with her [concerning cART and sexuality]?

Caregiver (mother): [I have to speak with her] about everything … everything.

Interviewer: And does that bother you?

Caregiver (mother): Yes all the time. Sometimes she says, “Oh mom you’re getting on my nerves.” I say, “Not everything is about getting on your nerves, because if I didn’t care about you I would just let you live your life.”

Conversely, some adolescents perceived that family members would not approve of conversations regarding sexual activity:

Female (1): Oh no, my aunt would kill me if I spoke to her about that.

Facilitator: Why?

Female (1): Because “That isn’t for your age” and this and that, or “You’re already interested in that?” and this and that. That’s what she says to me.

Female (1), 17 years.

Facilitator: You don’t have the trust [with your mom] to be able to discuss that topic [of sexuality]?

Male (1): No, I don’t even discuss my daily life with her.

Female [2]: My dad would kill me if I told him.

Male (1), 17; Female (2), 17 years.

#### Lack of information from alternative sources

Outside the home, adolescents reported having limited conversations regarding sexual education with knowledgeable adults. Some adolescents reported having no sexual education in school. Those who had described a lack of in-depth and practical information:

Facilitator: Tell me, in your school have they touched on the topic of protection or condom-use?

Male (2): […] they touch on the topic of STDs, STIs, and talk about condom use, but it isn’t … it is just to get it done. […] They bring it up, talk about it a little, and there it stays. You move on to the next topic. It isn’t very specific.

Male (2), 16 years.

Other adolescents reported receiving some condom-use education in the hospital or group home setting:

Facilitator: Have you had that opening, that confidence to be able to speak with someone where you are, in the group home?

Male (3): With the psychologist.

Facilitator: You have asked him?

Male (3): Yes.

Facilitator: And he already gave you guidance like what we have done here today?

Male (3): He speaks more about his opinions, he gives advice, he showed me how to [use a condom], but not with props.

Male (3), 16 years.

### Disclosure

**Fig. 3:**
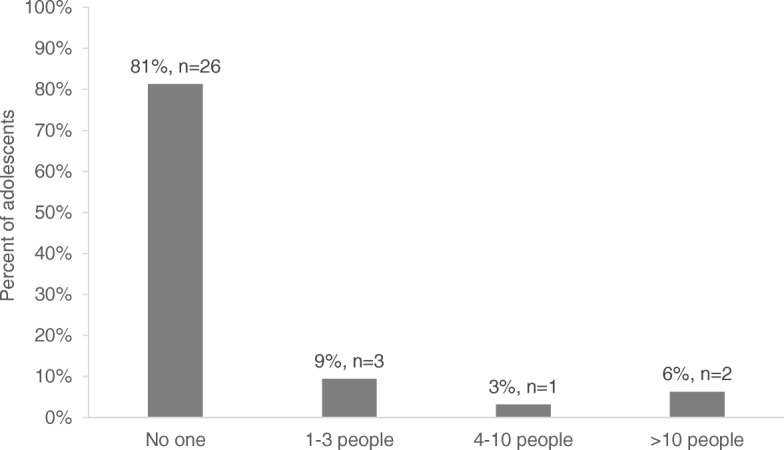
Number of people to whom adolescents disclosed HIV diagnosis (*N* = 32)

Most adolescents (*n* = 26, 81%) reported they had not disclosed their HIV diagnosis to any friends (Fig. 3). The mean age of adolescents who reported disclosing their diagnosis was 15.7 years (std 1.0). The peer counselor perceived that the degree of consanguinity was a determinant in disclosure. She described how knowledge of an HIV diagnosis is often limited to immediate family members:

[The patient’s] nuclear family, father, mother, brother/sister. They are the ones who know his/her diagnosis, who manage it. The grandmother, or the aunt who brings them [also knows]. But other family members, for example the brother-in-law, no, the cousin … no.

The health educator reported discouraging adolescents from sharing their diagnosis because reactions upon disclosure can be unpredictable:

Generally, we ask that they don’t do it. Because sometimes … in the long run, the only one who will suffer is [that individual]. Because not everyone understands this diagnosis, even if they are adults.

#### Disclosure to romantic partners

One support group session discussion focused on disclosure to romantic partners. Adolescents spoke about the importance of disclosing their diagnosis only to a romantic partner in whom they have confidence and an established relationship:

Male (4): I think that you can tell [a romantic partner about your condition] but first you have to know her well, no? The person that you are with.

Female (3): The girl with whom you’re getting involved … know that she is the right person more than anything.

Female (4): Because maybe you’re with someone and then suddenly the relationship ends after a week and the person takes it upon him/herself to tell everyone … your girlfriends, your guy friends, and sometimes, like in the case of some here … friends hadn’t known.

Male (4), 16 years; Female (3), 18 years; Female (4), 17 years.

The physician described that successful disclosure to romantic partners was rare:

We have other girls who are with one [sex partner] and then another. They have sex, and they are never going to say anything to these partners [about having HIV]. They won’t tell them even though we explain that it’s not right. Others have told their partners. I have several [patients] who have told their boyfriends--and are still with their boyfriends--who tell them there’s no problem, that they’ll support them, that they should make sure to take their medication. But these are few.

#### Reasons for disclosure

In spite of a fear of rejection, some adolescents still chose to disclose their diagnosis. Factors such as falling in love or reaching the new stage of a relationship influenced adolescents’ decisions to disclose to their partners:

Female (1): [I told my boyfriend] because I was already falling in love with him. And to keep lying to him and him liking me, I feel that would have been harmful to his life. That is why I told him. But we remained together, and he became well-informed [about HIV] …

[…]

Facilitator: So your idea was that he would leave you for another person, thinking that he would leave you because of your diagnosis?

Female (1): Yeah.

Female (1), 17 years.

Male (1): I was going to come live in [east-Lima] […] and I wanted a relationship, to keep having a relationship with her, but I wanted her to know [about my condition] so that she could decide if she wanted to stay with me or not. I wanted something serious with her, and I told her to see if she wanted something serious with me, too.

[…]

Male (1): I didn’t know how to tell her [about my HIV]. I was nervous.

Facilitator: But you did it.

Male (1): Yeah, I did.

Facilitator: You didn’t want to fall more in love [before you told her], because sometimes you fall in love and you are afraid that if she finds out she could leave you.

Male (1): For that very reason I wanted to tell her [now], because maybe I tell her and she no longer wants anything serious. Because of that, I told her.

Male (1), 16 years.

Clinicians described instances in which unprotected sexual encounters, followed by a fear of transmission, also prompted disclosure.

Nurse: A scared girl presented who had […] oral sex but had not used protection […]. Frightened, she said, “I infected him. How do I know if I’ve infected him?” [She was] worried because she hadn’t said anything [about her HIV] to the boy. [Because of this incident] the boy found out everything because she tells him. She said to me “I have attended all the [sexual health] talks and I listened, but my head was in another place. And once this happened I couldn’t remember if HIV could be transmitted through oral sex. So, it is difficult for them. It is difficult to face being adolescents.

Physician: I get a call in the early morning telling me, “I’ve told my boyfriend that I have HIV because we had oral sex and I didn’t know that oral sex could also transmit [HIV]. And now I want you to talk to him because he is freaking out and searching the internet.”

Health personnel described that among adolescents who have not disclosed their diagnosis, the efforts to keep their diagnosis concealed can negatively impact adherence. The psychologist described this connection:

There are lots of adherence problems. They get tired, or they enter the period of falling in love. They say, for example, “My girlfriend will find out what I am taking, she’s going to ask me why I am taking so many pills.” They say they can’t lie forever because, for example, they can say they have stomach problems … but there’s still the fear of being discovered. So, in the end, they abandon [cART].

### Substance use

**Fig. 4:**
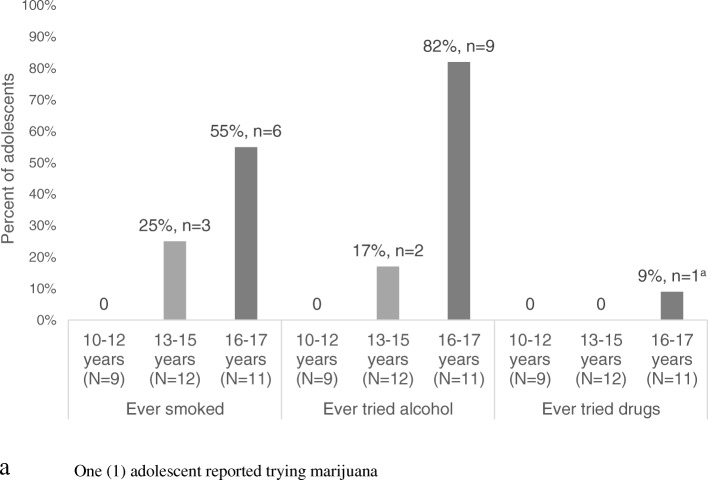
Percentage of adolescents reporting ever smoking cigarettes, trying alcohol, and trying drugs, by age group (*N* = 32)

In ACASI interviews, 34 and 3% of adolescents reported having tried alcohol and drugs, respectively (Table 1, Fig. 4). No adolescent in the 10–12 year age group reported having tried alcohol or drugs. The frequency of reported alcohol use increased among the older age groups with 82% of 16–17 year olds reporting having ever tried alcohol. While only one adolescent reported having ever tried drugs in ACASI interviews, transcriptions from psychosocial support groups suggested adolescents began experimenting with drugs, mainly marijuana, beginning at age 13. Adolescents that reported consistent use of drugs and/or alcohol generally contended that it did not affect their cART adherence:

Facilitator: And how did it go with your treatment at those times when you consumed [marijuana] … did you think of it? Did you not think of it?

Male (1): Well, my [consumption] was to experiment, nothing more.

Facilitator: And how did it go that day with your cART, did you take it?

Male (1): [I took it] as always. The group home made it a habit for me.

Male (1), 16 years.

## Discussion

We report health-related behaviors of ALHIV in Lima Peru. We found that cART non-adherence, substance use and unprotected sex begin to increase by ages 13–15 and are common through late adolescence. These results signal that targeted interventions–especially for managing romantic relationships–are needed early, before adolescents become sexually active and engage in risky behaviors.

A series of issues emerge when ALHIV reach sexual debut and begin romantic relationships. Adolescents can experience difficulty disclosing their HIV status to sexual partners due to fears of rejection, stigmatization, and public exposure [29–32]. As we found in this study, adolescents who have not disclosed may struggle with adherence and resort to hiding medications or discontinuing cART to maintain secrecy and avoid HIV-related stigma [29–34]. Ultimately, these adolescents are at risk of elevated viral load and increased likelihood of transmission, [35–37] compounded by low reported condom utilization in this age group [38]. While sexual debut and beginning romantic relationships are a distinguishing feature of adolescence, living with HIV contributes considerable additional stress in terms of disclosure and preventing HIV transmission. Interventions should respond to the unique needs of ALHIV by providing education on how to navigate romantic relationships when living with HIV, including disclosure to sexual partners, maintaining adherence, and contraceptive use.

We observed gaps in sexual health knowledge among adolescents. Surveys on HIV knowledge in Peru over the last decade have shown an upwards trend in awareness that condoms can prevent HIV [39]; however, adolescents in our study described a lack of knowledge regarding proper condom use. Sexual health knowledge may be lacking due to the absence of a compulsory, national sex curriculum implemented in schools [40]. Further, in qualitative interviews, adolescents and caregivers described hesitancy to discuss sex with one another. The use of trained peers educators or “escuela de padres” (“parent school”) initiatives, [40] where caregivers acquire knowledge and skills to engage in sexual health dialogue with their children, could help to disseminate important information on sexual health to adolescents. The need for instruction and understanding of safe sex practices and the hesitancy to discuss these topics with caregivers is likely generalizable to all adolescents, regardless of HIV status. However, ALHIV need additional education on how to prevent sexual transmission of HIV, either through use of condoms or virologic suppression (i.e., undetectable = untransmittable or “U=U” message) [41]. Health education for ALHIV, particularly around topics prone to misconception such as transmission, can be delivered in the clinic by health professionals. However, HIV programs are often operating in the backdrop of resource-constrained health systems and limited one-to-one interaction with health providers who can tend to adolescents’ medical and psychosocial needs. Thus, training peer educators and caregivers to deliver important health education messages could serve to fill this gap.

Provision of sexual health services, including condom distribution, is essential to prevent sexual transmission of HIV and unplanned pregnancy. We found that over half (56%) of sexually active adolescents reported having condomless sex during their last intercourse, comparable to the national average (64%) [38]. While we did not collect data on pregnancy in our study, national survey data suggest that pregnancy during adolescence is common, with 13% of women aged 15–19 years reporting having ever been pregnant [39]. In Peru, policies and norms surrounding provision of sexual health services to minors (< 18 years of age) without caregiver consent can dissuade providers from delivering family planning services [42]. This eliminates a critical point of care for adolescents who are reaching sexual debut as young as age 12. Local health policy that acknowledges that adolescents are sexually active and at risk of unprotected sex and responds by ensuring access to condoms and sexual health care is a key step to curtailing unwanted pregnancy during adolescence and transmission of sexually transmitted infections.

Substance use, which largely consisted of tobacco and alcohol, was common among older adolescents in our study. This finding is compatible with national data in which 60% of high school students reporting having used alcohol [38]. Qualitative data revealed that adolescents did not perceive alcohol or marijuana use to affect their adherence to cART. Although substance use was not perceived as a driver of nonadherence, drug and alcohol use have been shown to be associated with non-adherence among adults and adolescents in diverse settings, [43–45] and therefore it should be addressed by providers managing ALHIV and included in education interventions targeted to adolescents.

Study limitations include a small sample of adolescents who were selected based on physician referral for being at risk for cART non-adherence. While this inclusion criterion may limit generalizability to ALHIV without cART adherence risks, approximately one-third of all adolescents receiving care at the hospital from which participants were recruited met this criterion. While self-report is a simple and convenient method for measuring adherence and has been shown to correlate with immunologic and virologic outcomes across a variety of settings and age groups, [43] it may suffer from recall bias and social desirability bias [46]. We attempted to mitigate these biases by questioning on the previous 30-day period, reducing issues that may arise from lengthy recall periods. We also used the ACASI system, which does not require a face-to-face interview and may have reduced adolescent’s propensity to respond with answers they believe the interviewer would like to hear. Additionally, we did not assess caregiver education or sexual health knowledge, thus we could not comment on caregivers’ ability to provide accurate sexual health information to adolescents. Finally, qualitative data were collected through a psychosocial support group intervention and topics were driven by adolescents and not through a standard interview guide. Themes discussed during psychosocial support groups tended to align with topics in the health behaviors survey and included sexual and reproductive health, adherence, and disclosure. Despite these limitations, our study is distinctive in that much of the existing evidence on ALHIV comes from high-resource settings or settings of generalized epidemics [11, 12]. Thus, our findings reflect the experience of ALHIV in a low-resource, Latin American country and are likely generalizable to other countries in the region.

## Conclusions

HIV-positive adolescents in this setting reported substance use, sexual activity, and condom use that is characteristic for their age group and consistent with background rates. The interdependency of these health behaviors with non-disclosure to sexual partners and declines in cART adherence may contribute to poor clinical outcomes and secondary transmission of HIV. Multifactorial interventions dealing with disclosure, managing romantic relationships, improving sexual health knowledge, and developing strategies to sustain cART adherence throughout adolescence are urgently needed for this population and should begin in late childhood and early adolescence.

## Data Availability

The datasets used and/or analyzed during the current study are available from the corresponding author on reasonable request.

## Abbreviations

ACASI: audio computer-assisted self-interview
ALHIV: adolescents living with HIV
cART: combination antiretroviral therapy
INSN: Instituto Nacional de Salud del Niño

## References

[1] United Nations Children’s Fund. For every child, end AIDS – seventh stocktaking report, 2016. New York, 2016.

[2] United Nations Children’s Fund (UNICEF). Turning the tide against AIDS will require more concentrated focus on adolescents and young people 2017. Available at: https://data.unicef.org/topic/hivaids/adolescents-young-people/#.

[3] Jessor R (1991). Risk behavior in adolescence: a psychosocial framework for understanding and action. J Adolesc Health.

[4] Seinfeld J, Galarza F (2014). Understanding underage drinking in Peru: determinants of its frequency and intensity. Economia..

[5] World Health Organization. A Qualitative Review of Psychosocial Support Interventions for Young People Living with HIV. Geneva, Switzerland. 2009. WHO/FCH/CAH/ADH/09.05. Available at: http://apps.who.int/iris/bitstream/10665/70174/1/WHO_FCH_CAH_ADH_09.05_eng.pdf.

[6] Nichols SL, Bethel J, Garvie PA (2013). Neurocognitive functioning in antiretroviral therapy-naive youth with behaviorally acquired human immunodeficiency virus. J Adolesc Health.

[7] Fielden SJ, Sheckter L, Chapman GE (2006). Growing up: perspectives of children, families and service providers regarding the needs of older children with perinatally-acquired HIV. AIDS Care.

[8] Arrivillaga M, Martucci V, Hoyos PA, Arango A (2013). Adherence among children and young people living with HIV/AIDS: a systematic review of medication and comprehensive interventions. Vulnerable Child Youth Stud.

[9] Auld AF, Agolory SG, Shiraishi RW (2014). Antiretroviral therapy enrollment characteristics and outcomes among HIV-infected adolescents and young adults compared with older adults-seven African countries, 2004-2013. MMWR Morb Mortal Wkly Rep.

[10] Zanoni BC, Mayer KH (2014). The adolescent and young adult HIV cascade of care in the United States: exaggerated health disparities. AIDS Patient Care STDs.

[11] Johnson BT, Scott-Sheldon LA, Huedo-Medina TB, Carey MP (2011). Interventions to reduce sexual risk for human immunodeficiency virus in adolescents: a meta-analysis of trials, 1985-2008. Arch Pediatr Adolesc Med.

[12] Mavedzenge SN, Luecke E, Ross DA (2014). Effective approaches for programming to reduce adolescent vulnerability to HIV infection, HIV risk, and HIV-related morbidity and mortality: a systematic review of systematic reviews. J Acquir Immune Defic Syndr.

[13] United Nations Children’s Fund (UNICEF). Fast facts on Adolescents and Youth in Latin America and the Caribbean. Available at: http://www.unicef.org/media/files/Fast_facts_EN.doc.

[14] Shaw S, Amico KR (2016). Antiretroviral therapy adherence enhancing interventions for adolescents and young adults 13–24 years of age: a review of the evidence base. J Acquir Immune Defic Syndr.

[15] Slogrove AL, Schomaker M, Davies MA, Williams P, Balkan S, Ben-Farhat J (2018). The epidemiology of adolescents living with perinatally acquired HIV: a cross-region global cohort analysis. PLoS Med.

[16] Abadia-Barrero CE, Castro A (2006). Experiences of stigma and access to HAART in children and adolescents living with HIV/AIDS in Brazil. Soc Sci Med.

[17] Abadia-Barrero CE, Larusso MD (2006). The disclosure model versus a developmental illness experience model for children and adolescents living with HIV/AIDS in Sao Paulo, Brazil. AIDS Patient Care STDs.

[18] Hazra R, Stoszek SK, Freimanis Hance L, Pinto J, Marques H, Peixoto M (2009). Cohort profile: NICHD international site development initiative (NISDI): a prospective, observational study of HIV-exposed and HIV-infected children at clinical sites in Latin American and Caribbean countries. Int J Epidemiol.

[19] UNAIDS. AIDSinfo. Available at: http://aidsinfo.unaids.org/.

[20] Centro Nacional de Epidemiología, Prevención y Control de Enfermedades. Boletín Mensual, Diciembre 2018. Situación epidemiológica del VIH-Sida en el Perú. Available at: https://www.dge.gob.pe/portal/docs/vigilancia/vih/Boletin_2018/diciembre.pdf.

[21] United Nations Children’s Fund (UNICEF), Instituto Nacional de Estadística e Informática (INEI). Situation of children in Peru. Lima, Peru. 2008. Available at: https://www.unicef.org/peru/spanish/Folleto_ing_correc_1.pdf.

[22] Rogers SM, Willis G, Al-Tayyib A (2005). Audio computer assisted interviewing to measure HIV risk behaviours in a clinic population. Sex Transm Infect.

[23] Ghanem KG, Hutton HE, Zenilman JM, Zimba R, Erbelding EJ (2005). Audio computer assisted self interview and face to face interview modes in assessing response bias among STD clinic patients. Sex Transm Infect.

[24] Des Jarlais DC, Paone D, Milliken J (1999). Audio-computer interviewing to measure risk behaviour for HIV among injecting drug users: a quasi-randomised trial. Lancet..

[25] Centers for Disease Control and Prevention. 2015 Youth Risk Behavior Survey. Available at: www.cdc.gov/YRBSS.

[26] Pichon-Riviere E. The group process. Buenos Aires: New Vision; 1999.

[27] Dedoose. Version 7.0.23, web application for managing, analyzing, and presenting qualitative and mixed method research data (2016). Los Angeles, CA: SocioCultural Research Consultants, LLC (www.dedoose.com).

[28] Galea JT, Wong M, Muñoz M, Valle E, Leon SR, Díaz Perez D (2018). Barriers and facilitators to antiretroviral therapy adherence among Peruvian adolescents living with HIV: a qualitative study. PLoS One.

[29] Toska Elona, Cluver Lucie D., Hodes Rebecca, Kidia Khameer K. (2015). Sex and secrecy: How HIV-status disclosure affects safe sex among HIV-positive adolescents. AIDS Care.

[30] Mburu G, Hodgson I, Kalibala S (2014). Adolescent HIV disclosure in Zambia: barriers, facilitators and outcomes. J Int AIDS Soc.

[31] Hogwood J, Campbell T, Butler S (2013). I wish I could tell you but I can’t: adolescents with perinatally acquired HIV and their dilemmas around self-disclosure. Clin Child Psychol Psychiatry.

[32] Siu GE, Bakeera-Kitaka S, Kennedy CE, Dhabangi A, Kambugu A (2012). HIV serostatus disclosure and lived experiences of adolescents at the transition Clinic of the Infectious Diseases Clinic in Kampala, Uganda: a qualitative study. AIDS Care.

[33] Mutwa PR, Van Nuil JI, Asiimwe-Kateera B (2013). Living situation affects adherence to combination antiretroviral therapy in HIV-infected adolescents in Rwanda: a qualitative study. PLoS One.

[34] Calabrese SK, Martin S, Wolters PL, Toledo-Tamula MA, Brennan TL, Wood LV (2012). Diagnosis disclosure, medication hiding, and medical functioning among perinatally infected, HIV-positive children and adolescents. AIDS Care.

[35] Flynn PM, Rudy BJ, Douglas SD (2004). Virologic and immunologic outcomes after 24 weeks in HIV type 1-infected adolescents receiving highly active antiretroviral therapy. J Infect Dis.

[36] Gross R, Yip B, Re Iii VL, Wood E, Alexander CS, Harrigan PR (2006). A simple, dynamic measure of antiretroviral therapy adherence predicts failure to maintain HIV-1 suppression. J Infect Dis.

[37] Khan M, Song X, Williams K, Bright K, Sill A, Rakhmanina N (2009). Evaluating adherence to medication in children and adolescents with HIV. Arch Dis Child.

[38] Encuesta global de salud escolar. Resultados - Perú 2010/ Ministerio de Salud. Lima: MINSA, 2011. Available at: http://www.who.int/chp/gshs/GSHS_Report_Peru_2010.pdf.

[39] Instituto Nacional de Estadística e Informática (INEI) Perú Encuesta Demográfica y de Salud Familiar - ENDES 2014. Lima, Perú. Available at: https://www.inei.gob.pe/media/MenuRecursivo/publicaciones_digitales/Est/Lib1211/pdf/Libro.pdf.

[40] Motta A, Keough S, Prada E, et al., De la Normativa a la Práctica: la Política de Educación Sexual y su Implementación en el Perú. New York: Guttmacher Institute, 2017. Available at: https://www.guttmacher.org/es/report/politica-de-educacion-sexual-peru.

[41] Calabrese SK, Mayer KH (2019). Providers should discuss U=U with all patients living with HIV. Lancet HIV.

[42] Ministerio de Salud del Perú. Balance político normativo sobre el acceso de las y los adolescentes a los servicios de salud sexual, salud reproductiva y prevención del VIH-Sida Lima, Peru. 2009. Available at: http://bvs.minsa.gob.pe/local/MINSA/479_MINSA1414.pdf.

[43] Murphy DA, Belzer M, Durako SJ, Sarr M, Wilson CM, Muenz LR (2005). Longitudinal antiretroviral adherence among adolescents infected with human immunodeficiency virus. Arch Pediatr Adolesc Med.

[44] Hosek SG, Harper GW, Domanico R (2005). Predictors of medication adherence among HIV-infected youth. Psychol Health Med.

[45] Kim MH, Mazenga AC, Yu X (2017). High self-reported non-adherence to antiretroviral therapy amongst adolescents living with HIV in Malawi: barriers and associated factors. J Int AIDS Soc.

[46] Wagner G, Miller LG (2004). Is the influence of social desirability on patients' self-reported adherence overrated?. J Acquir Immune Defic Syndr.

